# Psychometric properties of the Arabic version of the Jaw Functional Limitation Scale (JFLS-Ar) in patients with temporomandibular disorders

**DOI:** 10.1186/s12903-025-07392-2

**Published:** 2025-12-09

**Authors:** Hosny Elkhawaga, Abdallah Gamiel, Mohamed Badr, Doaa Ahmed Haridy, Aya Abdelhamied Khalil

**Affiliations:** 1 Faculty of Physical Therapy, Cairo University, Giza, Egypt; 2 Department of Physical Therapy for Musculoskeletal Disorders and Its Surgery, Faculty of Physical Therapy, Modern University for Technology and Information (MTI) University, Cairo, Egypt; 3 Department of Physical Therapy for Neuromuscular Disorders and Its Surgery, Faculty of Physical Therapy, Al Hayah University in Cairo, Cairo, Egypt; 4 Department of Educational Psychology, Faculty of Graduate Studies of Education, Cairo University, Giza, Egypt; 5 Department of Biomechanics, Faculty of Physical Therapy, Cairo University, Giza, Egypt; 6 Faculty of Physical Therapy, Alamein International University, Alexandria, Egypt

**Keywords:** Temporomandibular disorders, Jaw functional limitation scale, JFLS, Patient-reported outcome measures, Psychometric properties

## Abstract

**Background:**

Temporomandibular disorders (TMDs) are a group of musculoskeletal conditions characterized by orofacial pain and functional impairments. The Jaw Functional Limitation Scale (JFLS) is a patient-reported outcome measure recommended by the Diagnostic Criteria for Temporomandibular Disorders. The aim of the study was to translate and cross-culturally adapt the JFLS for the Arabic language and to test its validity and reliability in Egyptian patients with TMDs.

**Methods:**

The JFLS was translated and cross-culturally adapted into Modern Standard Arabic according to standard guidelines. The construct validity was assessed in 54 patients with TMD who completed the Arabic versions of JFLS (JFLS-Ar), the Arabic version of Oral Health Impact Profile 5 (OHIP5-Ar), and the Numerical Pain Rating Scale (NPRS). Test–retest reliability was estimated in 30 participants who completed the JFLS-Ar again within seven days. Cronbach’s alpha coefficient was used to determine the internal consistency of the items in the JFLS-Ar.

**Results:**

The total score (0–200) of the JFLS-Ar showed moderate correlations with both OHIP5-Ar (ρ = 0.58, *p* < 0.001) and NPRS (ρ = 0.56, *p* < 0.001). The global score (average of the three domain scores, 0–10) showed moderate correlations with both OHIP5-Ar (ρ = 0.56, *p* < 0.001) and NPRS (ρ = 0.56, *p* < 0.001). The short form global score (average of 8 specific items, 0–10) showed moderate correlations with both OHIP5-Ar (ρ = 0.61, *p* < 0.001) and NPRS (ρ = 0.6, *p* < 0.001). The total score showed excellent test–retest reliability with an ICC of 0.97 (95% CI: 0.95–0.99), Standard Error of Measurement (SEM) of 6.17, and Minimal Detectable Change (MDC) of 17.1. The global score showed excellent test–retest reliability with an ICC of 0.97 (95% CI: 0.93–0.98), SEM of 0.38, and MDC of 1.06. The short form global score showed excellent test–retest reliability with an ICC of 0.99 (95% CI: 0.98–0.995), SEM of 0.2, and MDC of 0.55. The Cronbach’s alpha coefficient of the 20-item JFLS-Ar and the 8‐item JFLS-Ar was 0.938 and 0.852, respectively.

**Conclusions:**

The JFLS-Ar represents a valid and reliable instrument for use in Arabic-speaking patients with TMDs.

**Supplementary Information:**

The online version contains supplementary material available at 10.1186/s12903-025-07392-2.

## Background

Temporomandibular disorders (TMDs) are a group of musculoskeletal conditions characterized by orofacial pain and functional impairments in the temporomandibular joint and associated structures [1]. TMDs affect approximately 30% of the global population, with a higher prevalence among females [2, 3]. The etiology of TMDs is multifactorial, involving biomechanical, neuromuscular, bio-psychosocial, and biological factors [4, 5]. In addition to pain and functional limitations, TMDs can negatively affect sleep quality, overall quality of life, and oral health-related quality of life [6–8]. Diagnosis and classification of TMDs are challenging due to the variability and complexity of their clinical presentation. To address this issue, the Diagnostic Criteria for Temporomandibular Disorders (DC/TMD) was developed in 2014 and is now a globally accepted classification tool for the diagnosis of TMDs [9]. Notably, the International Network for Orofacial Pain and Related Disorders Methodology (INfORM) group of the International Association for Dental, Oral and Craniofacial Research (IADR) proposed a set of key points for good clinical practice in TMDs management, emphasizing patient-centered care, biopsychosocial etiology, standardized diagnostic procedures, and conservative treatment approaches [10].

Patient-reported outcome measures (PROMs) are recommended in patient management to support clinical decision-making [11]. PROMs can be used to evaluate the patient’s condition, assess the effects of interventions, monitor disease progression, and predict disease prognosis [12]. Several PROMs have been developed for the assessment of patients with TMDs [13]. The Jaw Functional Limitation Scale (JFLS) is a PROM recommended by the DC/TMD to assess functional limitations in patients with TMDs in both clinical and research settings [14, 15]. The JFLS has been cross-culturally adapted and validated in several languages, including Chinese [16], Malaysian English [17], Croatian [18], and Turkish [19]; however, no validated Arabic version is currently available. Therefore, the aim of the study was to translate and cross-culturally adapt the JFLS to the Modern Standard Arabic and to test its psychometric properties in Egyptian patients with TMDs.

## Methods

### Study design

This cross-sectional study consisted of two phases. First, the JFLS was translated and cross-culturally adapted from English to Modern Standard Arabic, which is the formal written form of Arabic used in education, healthcare, and research in Arabic-speaking countries [20]. Second, the validity and reliability of the final Arabic version were assessed. The study was approved by the Research Ethics Committee of the Faculty of Physical Therapy, Cairo University (No: P.T.REC\012\004655). Written informed consent was obtained from all participants.

### Study participants

The sample size was calculated for internal consistency and test–retest reliability to detect at least Cronbach’s Alpha Coefficient (α) of 0.7 (cut-off for Internal consistency) and intraclass correlation coefficient (ICC) of 0.5 (cut-off for moderate reliability). A minimum of 30 participants for Cronbach’s Alpha Coefficient and a minimum of 22 participants for ICC was recommended: (1) Cronbach’s Alpha Coefficient: number of items = 20, null hypothesis CA₀ = 0, expected α = 0.7 (2) ICC: observations = 2, null hypothesis R₀ = 0, expected ICC = 0.5, power = 80%, α = 0.05 [21, 22]. Participants of the study were patients with TMDs symptoms seeking care at the outpatient clinic of the Faculty of Physical Therapy, Cairo University. From April 2024 to January 2025, 54 individuals diagnosed with TMDs according to DC/TMD [9] were included in the study. Patients were classified into three different groups, including patients with myalgia, patients with internal derangement, and patients with subluxation, arthralgia, and degenerative joint disease [16]. Exclusion criteria were patients with neurological disorders, infection, and cancer.

### The Jaw functional Limitation Scale (JFLS)

The JFLS was first developed in 2008 and consists of 20 items that assess functional limitation in patients with TMDs [14, 15]. Each item is scored on an 11-point scale (0 = no limitation, 10 = severe limitation), with a total score ranging from 0 to 200 [23]. Higher scores indicate greater functional impairment. The JFLS includes three domains: the mastication domain (Items 1–6), the vertical jaw mobility domain (Items 7–10), and the verbal and emotional expression domain (Items 13–20). Each domain score (ranging from 0 to 10) is calculated by averaging the item scores within that domain. A global score (0–10) is also calculated by averaging the three domain scores. Items 11 (swallowing) and 12 (yawning) are not included in domain or global scoring, as these items assess swallowing and yawning limitations separately [19, 24].

In addition to the full version, a short-form version of the JFLS has been developed, comprising 8 items (items 1, 3, 6, 10–13, and 19) from the full 20-item scale. The global score (0–10) of the short-form version is calculated as the average of these 8 item scores [15, 24]. The psychometric properties of the short-form version of the Arabic versions of JFLS (JFLS-Ar) were additionally investigated in the study.

### Translation and cross‑cultural adaptation

Translation and cross-cultural adaptation of the JFLS to the Arabic language followed the ISPOR guidelines [25]. First, two independent professional bilingual translators with no medical background forwardly translated the original version of the JFLS into Arabic, resulting in two forward-translated versions (F1 and F2). Second, the two versions were reviewed and reconciled into a third initial Arabic version (F3) using a consensus-based approach. The consensus process was performed by the research team, which included three physical therapists specialized in TMDs (A.G., M.B., and H.E.), a linguistic reviewer (D.H.), and a senior academic supervisor (A.A.K.). During the reconciliation phase, a minor cultural adjustment was made to enhance relevance. In item 6, “apple sauce” was removed due to limited familiarity in Arabic-speaking population, and “pudding” was clarified as “jelly/pudding”. Third, two other independent bilingual translators who were blinded to the original version of the JFLS performed back translation into English (B1 and B2) of the (F3) version. Fourth, the two back-translated versions (B1 and B2) were compared to the original version of the JFLS regarding items, meaning, sentence structure, relevance, and wording. Based on this review, the reconciled Arabic version was considered the pre-final version of the scale (pre-final JFLS-Ar).

Fifth, cognitive debriefing was conducted with six bilingual participants diagnosed with TMDs, including two dentists, one physical therapist, and three other individuals fluent in both Arabic and English. Each participant completed both the original English version of the JFLS and the pre-final JFLS-Ar. Then, they were asked to evaluate each item of the pre-final JFLS-Ar and interviewed regarding their understanding of each item in both versions, and asked to identify any unclear or inconsistent wording. No major issues were reported during the interviews, and minor adjustments were made for clarity. For testing the equivalency, a Wilcoxon signed-rank test was conducted to compare the participants’ responses to the original English version and the pre-final JFLS-Ar. Finally, proofreading was done by a linguistic reviewer for the pre-final version to finalize it. Consequently, the final version of the JFLS-Ar was prepared and used for psychometric testing in the second phase of the study.

### Construct validity testing

At the first testing session, participants were asked to fill the Arabic-translated questionnaires including: (1) the final version of JFLS-Ar; (2) the Arabic version of Oral Health Impact Profile 5 (OHIP5-Ar) [26], which is a valid and reliable instrument for assessing oral health-related quality of life [27, 28]. The OHIP5-Ar consists of five items rated on a 5-point Likert scale (0 = never; 1 = hardly ever; 2 = occasionally; 3 = fairly often; 4 = very often), with a total score ranging from 0 to 20, where higher scores indicate worse oral health-related quality of life. The OHIP5-Ar was chosen because it has been tested and validated in German and Dutch TMDs patients [28, 29] and it is currently the only available Arabic-language questionnaire suitable for assessing oral health-related quality of life in TMDs patients.; (3) The Numerical Pain Rating Scale (NPRS) that measures current pain intensity between 0 (no pain) and 10 (maximum possible pain). Construct validity was evaluated by correlating the scores of the JFLS-Ar and the OHIP5-Ar and NPRS using the Spearman rank correlation coefficients (*ρ*). The strength of correlation was described as negligible (0.00–0.10), weak (0.10–0.39), moderate (0.40–0.69), strong (0.70–0.89), and very strong (0.90–1.00) [30].

### Reliability testing

Participants were instructed to refill the JFLS-Ar for the second time within seven days. Out of the 54 participants, 30 completed the JFLS-Ar for the second time and were included in the test–retest reliability analysis. The test-retest reliability of the JFLS-Ar between the first and second times was tested by calculating the intraclass correlation coefficient (ICC; Two-way mixed effects model (absolute agreement)) with corresponding 95% confidence intervals (CI) [31]. An ICC value > 0.90 was considered indicative of excellent reliability, values between 0.75 and 0.90 indicated good reliability, values between 0.50 and 0.75 indicated moderate reliability, and values < 0.50 indicated poor reliability [31]. The Standard Error of Measurement (SEM), which is the square root of the error variance of the ANOVA analysis, was calculated to determine the absolute measurement error [32]. Additionally, the SEM was used to calculate the Minimal Detectable Change (MDC), which represents the changes that fall outside the measurement error and equals 1.96 × √2 × SEM [33]. Internal consistency between items was assessed using Cronbach’s Alpha Coefficient (α), with a value ≥ 0.70 considered acceptable [34].

### Statistical analysis

The basic demographics were presented as mean ± standard deviation (SD) or frequency (percentage). IBM SPSS version 27 (IBM Corp., Armonk, USA) was used for demographic data, validity, and reliability analysis, with statistical significance being accepted with a p-value < 0.05.

## Results

### Equivalency between the english version and the pre-final JFLS-Ar

There was no significant difference between the total scores of the original English version and the pre-final JFLS-Ar (*P* = 0.686), indicating that the pre-final JFLS-Ar was equivalent to the original version.

### Translation and cultural adaptation process

During the cognitive debriefing, all participants indicated that the pre-final JFLS-Ar items were clear and easy to understand. Cultural relevance was assessed during the cognitive debriefing phase. Participants confirmed that all items, including those related to diet (e.g., soft food examples) and social activities, were understandable, applicable, and culturally appropriate within the Arabic-speaking population. Based on their feedback, minor adjustments were made to improve clarity. These included: (1) adding diacritical marks to items 7, 8, 9, 10, 15, and 17 to enhance readability and pronunciation; (2) applying gender adaptation in items 15 and 16 by adding the feminine form to ensure inclusiveness and cultural appropriateness; and (3) adding a synonym in parentheses in item 17 to clarify the meaning of (frowning). These modifications led to the final version of the JFLS-Ar (Appendix 1).

### Study participants

Fifty-four patients with TMDs were included in this study. Their mean age (± SD) was 29.69 (± 11.36) years, and 39/54 (72.22%) were female. The demographic characteristics of the participants are summarized in Table 1.

**Table 1:** Demographic characteristics of the participants (*n* = 54)

Characteristics	Descriptive statistics
Age (years)	29.69 ± 11.36
Height (cm)	69.13 ± 11.69
Weight (kg)	166.24 ± 8.34
BMI (kg/m^2^)	25.07 ± 4.3
Gender, n (%)	
Male	15 (27.78)
Female	39 (72.22)
Diagnosis according to DC/TMD, n (%)	
Myalgia	21 (38.89)
Internal derangement	23 (42.59)
Subluxation, arthralgia, and degenerative joint disease	10 (18.52)
Symptom duration (months), n (%)	
≤ 3 months	10 (18.5)
> 3 to ≤ 6 months	11 (20.4)
> 6 to ≤ 12 months	11 (20.4)
> 12 to ≤ 24 months	9 (16.7)
> 24 months	13 (24.1)
Mean ± SD	19.98 ± 24.53
OHIP5	7.67 ± 4.51
NPRS	4.93 ± 2.3

*DC/TMD* Diagnostic Criteria for Temporomandibular Disorders, *NPRS* Numerical Pain Rating Scale, *OHIP5-Ar* Arabic version of Oral Health Impact Profile 5

### Construct validity

Moderate correlation coefficients were observed for the relationships between the JFLS-Ar total score (1–200) and both the OHIP5-Ar (ρ = 0.58, *p* < 0.001) and the NPRS (ρ = 0.56, *p* < 0.001). Regarding domains, the mastication domain showed moderate correlations with the OHIP5-Ar (ρ = 0.50, *p* < 0.001) and the NPRS (ρ = 0.60, *p* < 0.001). The vertical jaw mobility domain demonstrated similar moderate correlations with OHIP5-Ar (ρ = 0.47, *p* < 0.001) and the NPRS (ρ = 0.50, *p* < 0.001). The verbal and emotional expression domain showed moderate correlation with the OHIP5-Ar (ρ = 0.63, *p* < 0.001) and weak correlation with the NPRS (ρ = 0.39, *p* < 0.001). The global score (average of the three domain scores, 0–10) showed moderate correlations with both OHIP5-Ar (ρ = 0.56, *p* < 0.001) and NPRS (ρ = 0.56, *p* < 0.001). The short form global score (average of 8 specific items, 0–10) showed moderate correlations with both OHIP5-Ar (ρ = 0.61, *p* < 0.001) and NPRS (ρ = 0.6, *p* < 0.001). The Spearman rank correlation coefficients (ρ) of the JFLS-Ar with the OHIP5-Ar and the NPRS are presented in Table 2.

**Table 2:** Spearman rank correlation coefficients (ρ) of the JFLS-Ar with the OHIP5-Ar and the NPRS (*n* = 54)

	JFLS-Ar total score (0–200)		Mastication domain (0–10)		Vertical jaw mobility domain (0–10)		Verbal and emotional expression domain (0–10)		Global score (0–10)		Short form global score (0–10)	
	ρ	p	ρ	p	ρ	p	ρ	p	ρ	p	ρ	p
OHIP5-Ar	0.58	< 0.001	0.5	< 0.001	0.47	< 0.001	0.63	< 0.001	0.56	< 0.001	0.61	< 0.001
NPRS	0.56	< 0.001	0.6	< 0.001	0.5	< 0.001	0.39	< 0.001	0.56	< 0.001	0.6	< 0.001
Mean ± SD	51.89 ± 38.93		3.01 ± 2.36		3.78 ± 2.85		1.53 ± 2.03		2.77 ± 2.088		2.61 ± 1.97	

*JFLS-Ar *Arabic version of Jaw Functional Limitation Scale, *NPRS *Numerical Pain Rating Scale, *OHIP5-Ar* Arabic version of Oral Health Impact Profile 5

### Reliability

For test-retest reliability, 30 patients re-filled the JFLS-Ar for the second time. The total score (0–200) of the JFLS-Ar showed excellent test–retest reliability with an ICC of 0.97 (95% CI: 0.95–0.99), SEM of 6.17, and MDC of 17.1. The mastication domain has excellent test–retest reliability with an ICC of 0.91 (95% CI: 0.82–0.96), SEM of 0.68, and MDC of 1.89. The vertical jaw mobility domain has good reliability with an ICC of 0.85 (95% CI: 0.70–0.92), SEM of 1.05, and MDC of 2.9. The verbal and emotional expression domain showed excellent reliability with an ICC of 0.95 (95% CI: 0.90–0.98), SEM of 0.51, and MDC of 1.43. The global score (average of the three domain scores, 0–10) demonstrated excellent test–retest reliability with an ICC of 0.97 (95% CI: 0.93–0.98), SEM of 0.38, and MDC of 1.06. The short form global score (average of 8 specific items, 0–10) has excellent test–retest reliability with an ICC of 0.99 (95% CI: 0.98–0.995), SEM of 0.2, and MDC of 0.55. Table 3 indicates the test–retest reliability results for each JFLS-Ar item, total score, domain scores, and global score.

**Table 3:** Test–retest reliability results for each JFLS-Ar item, total score, domain scores, and global score (*n* = 30)

JFLS-Ar item	Mean ± SD (first test)	Mean ± SD (re-test)	ICC (95% CI)	SEM	MDC
1. Chew tough food (0–10)	4.77 ± 3.33	4.8 ± 3.19	0.96 (0.92–0.98)	0.63	1.74
2. Chew hard bread (0–10)	4.9 ± 3.35	4.93 ± 3.33	0.97 (0.95–0.99)	0.54	1.5
3. Chew chicken (0–10)	3.07 ± 3.21	2.9 ± 2.94	0.96 (0.92–0.98)	0.59	1.63
4. Chew crackers (0–10)	1.87 ± 2.45	1.93 ± 2.59	0.74 (0.51–0.86)	1.31	3.64
5. Chew soft food (0–10)	1.37 ± 2.17	1.53 ± 2.61	0.59 (0.3–0.78)	1.54	4.28
6. Eat soft food requiring no chewing (0–10)	0.33 ± 1.03	0.7 ± 1.93	0.43 (0.09–0.68)	1.17	3.23
7. Open wide enough to bite from a whole apple (0–10)	6.8 ± 3.61	5.57 ± 3.43	0.87 (0.75–0.94)	1.28	3.55
8. Open wide enough to bite into a sandwich (0–10)	4.07 ± 3.73	4.87 ± 3.55	0.86 (0.73–0.93)	1.36	3.76
9. Open wide enough to talk (0–10)	2.7 ± 3.33	2.27 ± 2.88	0.83 (0.68–0.92)	1.27	3.52
10. Open wide enough to drink from a cup (0–10)	1.6 ± 2.91	1.7 ± 2.67	0.91 (0.82–0.96)	0.84	2.32
11. Swallow (0–10)	0.97 ± 1.97	0.9 ± 2.02	0.97 (0.93–0.98)	0.37	1.02
12. Yawn (0–10)	5.77 ± 3.35	5.57 ± 3.31	0.95 (0.9–0.98)	0.73	2.02
13. Talk (0–10)	1.87 ± 2.78	1.67 ± 2.68	0.88 (0.77–0.94)	0.94	2.59
14. Sing (0–10)	1.67 ± 2.89	1.8 ± 2.98	0.94 (0.87–0.97)	0.74	2.04
15. Putting on a happy face (0–10)	1.37 ± 2.37	1.27 ± 2.39	0.89 (0.78–0.95)	0.8	2.2
16. Putting on an angry face (0–10)	1.17 ± 2.42	1.3 ± 2.58	0.98 (0.86–0.99)	0.32	0.88
17. Frown (0–10)	0.73 ± 2.24	0.87 ± 2.18	0.86 (0.72–0.93)	0.85	2.34
18. Kiss (0–10)	0.80 ± 2.25	0.93 ± 2.32	0.83 (0.68–0.92)	0.94	2.61
19. Smile (0–10)	1.47 ± 2.68	1.33 ± 2.45	0.96 (0.92–0.98)	0.52	1.43
20. Laugh (0–10)	2.4 ± 2.97	2.3 ± 2.79	0.94 (0.87–0.97)	0.73	2.02
Total Score (0–200)	49.67 ± 39.3	49.13 ± 38.71	0.97 (0.95–0.99)	6.17	17.1
Mastication domain (0–10)	2.72 ± 2.13	2.8 ± 2.35	0.91 (0.82–0.96)	0.68	1.89
Vertical jaw mobility domain (0–10)	3.79 ± 2.81	3.6 ± 2.47	0.85 (0.7–0.92)	1.05	2.9
Verbal and emotional expression domain (0–10)	1.43 ± 2.23	1.43 ± 2.23	0.95 (0.9–0.98)	0.51	1.43
Global score (0–10)	2.65 ± 2.07	2.61 ± 1.98	0.97 (0.93–0.98)	0.38	1.06
Short form global score (0–10)	2.48 ± 1.93	2.45 ± 1.88	0.99 (0.98–0.995)	0.2	0.55

*ICC* Intraclass correlation coefficient, *JFLS-Ar* Arabic version of Jaw Functional Limitation Scale, *MDC* Minimal Detectable Change, *SEM* Standard Error of Measurement

Regarding internal consistency, the Cronbach’s alpha coefficient of the 20-item JFLS-Ar and the 8‐item JFLS-Ar was 0.938 and 0.852, respectively. The Cronbach’s alpha coefficient of the mastication domain, the vertical jaw mobility domain, and the verbal and emotional expression domain was 0.904, 0.847, and 0.914, respectively. The Cronbach’s alpha coefficient for the 18 items assessing global limitation was 0.937. The correlation between items and the Cronbach’s alpha if items were deleted for the 20‐item JFLS-Ar are shown in Table 4.

**Table 4:** Internal consistency of the JFLS-Ar (*n* = 54)

JFLS-Ar item	Corrected Item-Total Correlation	Cronbach’s Alpha if Item Deleted
1. Chew tough food	0.728	0.933
2. Chew hard bread	0.727	0.933
3. Chew chicken	0.72	0.933
4. Chew crackers	0.671	0.934
5. Chew soft food	0.719	0.934
6. Eat soft food requiring no chewing	0.583	0.936
7. Open wide enough to bite from a whole apple	0.588	0.936
8. Open wide enough to bite into a sandwich	0.608	0.936
9. Open wide enough to talk	0.708	0.933
10. Open wide enough to drink from a cup	0.766	0.932
11. Swallow	0.434	0.938
12. Yawn	0.537	0.937
13. Talk	0.747	0.933
14. Sing	0.644	0.934
15. Putting on a happy face	0.6	0.935
16. Putting on an angry face	0.593	0.935
17. Frown	0.591	0.936
18. Kiss	0.721	0.934
19. Smile	0.634	0.935
20. Laugh	0.646	0.934

*JFLS-Ar* Arabic version of Jaw Functional Limitation Scale

## Discussion

In this study, JFLS was successfully translated and cross-culturally adapted into Modern Standard Arabic. The JFLS-Ar demonstrated acceptable psychometric properties regarding validity and reliability. This supports its use in both clinical and research contexts for Arabic-speaking patients with TMDs.

Convergent validity was examined to assess the construct validity of the JFLS-Ar. Moderate positive correlations were found between the JFLS-Ar total score and both the OHIP5-Ar (ρ = 0.58) and NPRS (ρ = 0.56). Similarly, moderate positive correlations were between the JFLS-Ar global score and both the OHIP5-Ar (ρ = 0.56) and NPRS (ρ = 0.56). This indicates that greater jaw functional limitation in patients with TMDs is associated with poorer oral health-related quality of life and higher levels of perceived pain. Regarding domains, the mastication domain (ρ = 0.50), vertical jaw mobility domain (ρ = 0.47), and verbal and emotional expression domain (ρ = 0.63) demonstrated moderate correlations with the OHIP5-Ar. The strongest correlation was observed in the verbal and emotional expression domain, suggesting that limitations in this domain may have more impact on patients’ oral health-related quality of life. Moreover, the mastication domain (ρ = 0.60), vertical jaw mobility domain (ρ = 0.50), and verbal and emotional expression domain (ρ = 0.39) demonstrated moderate to weak positive correlations with the NPRS. The strongest correlation was in the mastication domain, suggesting that greater difficulty in mastication activities is associated with higher levels of perceived pain in patients with TMDs. Conversely, the verbal and emotional expression domain showed the weakest correlation with pain. This suggests that although functional limitation in this domain has more impact on oral health-related quality of life, it may be less associated with pain intensity. These findings suggest that functional limitation in JFLS-Ar domains may variably reflect oral health-related quality of life and pain perception in patients with TMDs.

The findings of our study regarding construct validity are in line with previous validation studies conducted in Chinese [16], Malaysian English [17], Croatian [18], and Turkish [19] versions of the JFLS, which also demonstrated moderate to strong correlations between the JFLS scores and both pain intensity and oral health-related quality of life measured by OHIP-22 and OHIP-14. In the Malaysian English version [17], the JFLS total score showed a strong correlation with oral health-related quality of life measured by OHIP-22 (ρ = 0.75), which is higher than the correlation found in our Arabic version (ρ = 0.58). Furthermore, the Turkish version [19] showed the strongest correlations across domains with the OHIP-14, with correlation coefficients reported for the mastication domain (ρ = 0.86), vertical jaw mobility domain (ρ = 0.81), and verbal and emotional expression domain (ρ = 0.76). These correlations are higher than the corresponding correlations in our Arabic version (ρ = 0.50, 0.47, and 0.63, respectively). The global score in the Turkish version also showed a strong correlation with OHIP-14 (ρ = 0.82), compared to a moderate correlation in our Arabic version (ρ = 0.56). Regarding pain intensity, the Chinese version [16] showed a weak correlation between the JFLS total score and pain intensity (*r* = 0.314), which is lower than the moderate correlation in our Arabic version (ρ = 0.56). Conversely, the Turkish version [19] showed the strongest correlations across domains with NPRS, with correlation coefficients reported for the mastication domain (ρ = 0.90), vertical jaw mobility domain (ρ = 0.87), and verbal and emotional expression domain (ρ = 0.80) domains, as well as the global score (ρ = 0.87). These values are higher than those reported in our Arabic version (ρ = 0.60, 0.50, 0.39, and 0.56, respectively). The observed variations in correlation coefficients between the Arabic version of the JFLS and other language versions may be attributed to several factors. Cultural differences could influence how patients express pain and disability, as some populations may underreport or tolerate symptoms more than others. Additionally, Variations in OHIP versions (OHIP-5, OHIP-14, and OHIP-22) used for assessment of oral health-related quality of life and differences in sample characteristics such as age, gender, severity and type of TMDs may also contribute to variability in correlations.

The test–retest reliability of the JFLS-Ar total score was excellent (ICC = 0.97), which is in line with that of the Malaysian English version (ICC = 0.97) [17] and slightly higher than that of the Chinese version (ICC = 0.871) [16]. Similarly, the JFLS-Ar global score demonstrated excellent reliability (ICC = 0.97), which is comparable to the Turkish version (ICC = 0.95) [19]. Moreover, the mastication, vertical jaw mobility, and verbal and emotional expression domains showed ICCs of 0.91, 0.85, and 0.95, respectively. These values are comparable to those mentioned in the Turkish version (0.94, 0.91, and 0.92) [19] and the Malaysian English version (0.98, 0.97, and 0.92) [17]. The MDC of the JFLS-Ar total score and global score was 17.1 and 1.06, respectively, which is in line with the global score MDC in the Turkish version (0.79) [19]. For the domains, the mastication, vertical jaw mobility, and verbal and emotional expression domains showed MDCs of 1.89, 2.9, and 1.43, respectively. These values are slightly larger than those documented in the Turkish version (1.43, 1.39, and 1.29, respectively) [19].

Regarding internal consistency, the Cronbach’s alpha coefficient was found to be 0.938 for the 20-item JFLS-Ar, which is comparable to the Malaysian English (α = 0.96) [17], Turkish (α = 0.93) [19], and Chinese (α = 0.906) [16] versions. Moreover, the mastication, vertical jaw mobility, and verbal and emotional expression domains showed Cronbach’s alpha coefficients of 0.904, 0.847, and 0.914, respectively. These values are consistent with those exhibited in the Turkish version (α = 0.91, 0.92, and 0.91, respectively) [19] and the Croatian version (α = 0.88, 0.76, and 0.91, respectively) [18]. Regarding the 18 items assessing global limitation of the JFLS-Ar, the Cronbach’s alpha coefficient was 0.937, which is similar to that of the Croatian version (α = 0.93) [18].

In addition to the full version, the psychometric properties of the 8-item short form of the JFLS-Ar were additionally assessed in the study. Moderate positive correlations were found between the 8-item short form of the JFLS-Ar score and both the OHIP5-Ar (ρ = 0.61) and NPRS (ρ = 0.6). The test–retest reliability was excellent (ICC = 0.97), with an MDC of 0.55. Regarding internal consistency, the Cronbach’s alpha coefficient was 0.852, which is comparable to the Croatian version (α = 0.81) [18]. Overall, the findings of this study revealed the validity and reliability of the full version and the 8-item short form of the JFLS-Ar and indicate that the JFLS-Ar provides stable and consistent measurement, which is in line with previous studies conducted with different languages [16–19].

According to the recent INfORM/IADR consensus on good clinical practice [10], TMDs are considered as multifactorial biopsychosocial disorder. These recommendations provide a contemporary framework that complements the DC/TMD classification and supports evidence-based clinical decision-making [9]. The DC/TMD represents a standardized, evidence-based approach for assessing TMDs across clinical and research settings. It is structured around two complementary axes. Axis I focuses on physical diagnoses derived from clinical examination, including muscle disorders, joint disorders, and disc displacements. Axis II addresses biopsychosocial evaluation of jaw function, behavioral and psychosocial factors; including pain-related disability, psychological distress, jaw functional limitation, and oral behaviors.

Jaw functional limitation assessment, using the JFLS in both its short (JFLS-8) and long (JFLS-20) forms, provides a structured, patient-reported measure of jaw-related disability [14, 15]. These tools enable clinicians and researchers to quantify limitations in mastication, vertical jaw mobility, and verbal and emotional expression. This may guide treatment goals and monitoring treatment progress. Therefore, translating the JFLS into Arabic enhances its clinical and research utility across Arabic-speaking populations. In addition to jaw functional limitation assessment, Axis II provides psychological instruments including the Patient Health Questionnaire-4 [35], Patient Health Questionnaire-9 [36], Patient Health Questionnaire-15 [37], and Generalized Anxiety Disorder-7 [38]. Evaluation of the psychological aspects in patients with TMDs is essential, as depression is both a risk factor for developing TMDs and highly prevalent among those affected [39, 40]. Additionally, several studies have reported a significant association between pain-related disability and elevated levels of depression and somatization among patients with TMDs [41–44]. Moreover, psychosocial findings have been shown to significantly predict treatment outcomes in TMDs patients [45]. Axis II also includes the Oral Behaviors Checklist (OBC), a validated self-report instrument designed to assess parafunctional oral activities such as bruxism (both sleep and awake), jaw clenching, teeth contact without chewing, tongue pressing, and other non-functional oral habits. These behaviors are considered potential contributing factors to the onset and persistence of TMDs [46, 47]. Bruxism is a parafunctional oral behavior associated with psychological distress [48–50]. The Standardised Tool for the Assessment of Bruxism (STAB) is a multidimensional instrument designed to evaluate bruxism status, comorbid conditions, aetiology and consequences [51, 52]. The integration of these instruments enables a comprehensive, multidimensional evaluation of patients with TMDs. This approach facilitates the identification of interrelated biopsychosocial factors, supports individualized treatment planning, and allows for monitoring clinical progress over time. This is fully aligned with the INfORM/IADR consensus, which emphasizes the multifactorial nature of TMDs [10].

Regarding strength points and limitations of this study, the translation and cultural adaptation of the JFLS-Ar followed the ISPOR guidelines [25], ensuring methodological rigor through independent forward and back translations, expert consensus, cognitive debriefing, and statistical equivalency testing. This approach aligns with recent translation frameworks used for newly introduced instruments, such as the 12-step guideline adopted in the STAB translation by Colonna et al., 2024 [53] and in OBC translation by Reda et al., 2024 [54], which emphasizes clarity, cultural relevance, and psychometric integrity. The psychometric evaluation included the total score, domain scores, and global score of the JFLS-Ar. Additionally, the psychometric properties of the 8-item short form of the JFLS-Ar were assessed in this study. Although convergent validity, test–retest reliability, and internal consistency of the JFLS-Ar were comprehensively evaluated, the study did not investigate other aspects of construct validity (e.g., discriminant or known-group validity) or responsiveness. Therefore, future research is recommended to explore these psychometric properties. While the JFLS-Ar was translated using Modern Standard Arabic to ensure broad applicability across Arabic-speaking populations, we acknowledge that regional dialects and cultural nuances may still influence item interpretation. Therefore, we recommend that future research consider either country-specific linguistic adaptations or a pan-Arab harmonized version that incorporates feedback from multiple Arabic-speaking regions to enhance cross-cultural adaptability. Moreover, although the sample size met the minimum requirements for evaluating reliability and internal consistency, it remains relatively small for broader psychometric generalization. Therefore, this study should be considered a pilot validation of the JFLS-Ar, and future research with larger and more diverse samples is recommended to confirm its structural validity and generalizability. Due to the biopsychosocial nature of TMD [10] and the established association between bruxism and psychological distress [48–50], further research is recommended to evaluate JFLS-Ar scores in populations with bruxism in the form of bracing. This may help clarify the functional impact of bracing behaviors and their role in mediating the relationship between psychological factors and jaw-related disability.

## Conclusion

The JFLS-Ar demonstrated acceptable psychometric properties and can be used as a valid and reliable tool to assess jaw functional limitation in Arabic-speaking patients with TMDs in research and clinical practice.

## Supplementary Information

Supplementary Material 1.

## Data Availability

The data associated with the paper are not publicly available but are available from the corresponding author on reasonable request.

## Abbreviations

CI: Confidence interval
DC/TMD: Diagnostic Criteria for Temporomandibular Disorders
IADR: International Association for Dental, Oral and Craniofacial Research
ICC: Intraclass correlation coefficient
INfORM: International Network for Orofacial Pain and Related Disorders Methodology
JFLS: Jaw Functional Limitation Scale
JFLS-Ar: Arabic version of the Jaw Functional Limitation Scale
MDC: Minimal Detectable Change
NPRS: Numerical Pain Rating Scale
OBC: Oral Behaviors Checklist
OHIP5-Ar: Arabic version of Oral Health Impact Profile 5
PROMs: Patient-reported outcome measures
SEM: Standard Error of Measurement
STAB: Standardised Tool for the Assessment of Bruxism
TMDs: Temporomandibular disorders
