# Supplementary material for: Psychometric properties of the Arabic version of the Jaw Functional Limitation Scale (JFLS-Ar) in patients with temporomandibular disorders
Source: BMC Oral Health. 2025 Dec 9;26:207. doi: 10.1186/s12903-025-07392-2 (PMC12860105; doi:10.1186/s12903-025-07392-2)
Supplement: Supplementary file 1 — Supplementary Material 1. [file 12903_2025_7392_MOESM1_ESM.docx]

**مقياس المدى الوظيفي للفك – 20**

أمامك مجموعة من الأنشطة/الأفعال. يرجى توضيح إلى أي مدى **خلال الشهر الماضي** تم تجنب النشاط/الفعل. فإذا تم تجنب النشاط/الفعل تماماً لأنه صعب للغاية عليك، ضع دائرة على الرقم (10). أما إذا تم تجنب النشاط/الفعل لأسباب أخرى غير الألم أو مستوى الصعوبة في الأداء، فاتركه دون الإجابة عليه.

| أواجه صعوبة شديدة | |  |  |  |  |  |  |  | لا أواجه صعوبة | |  |
| --- | --- | --- | --- | --- | --- | --- | --- | --- | --- | --- | --- |
| 10 | 9 | 8 | 7 | 6 | 5 | 4 | 3 | 2 | 1 | 0 | 1. مضغ الطعام الناشف |
| 10 | 9 | 8 | 7 | 6 | 5 | 4 | 3 | 2 | 1 | 0 | 2. مضغ الخبز الناشف |
| 10 | 9 | 8 | 7 | 6 | 5 | 4 | 3 | 2 | 1 | 0 | 3. مضغ الدجاج (على سبيل المثال، المُجهز في الفرن) |
| 10 | 9 | 8 | 7 | 6 | 5 | 4 | 3 | 2 | 1 | 0 | 4. مضغ البسكويت |
| 10 | 9 | 8 | 7 | 6 | 5 | 4 | 3 | 2 | 1 | 0 | 5. مضغ الطعام الطري (مثل المكرونة والفواكه المعلبة أو الطرية والخضروات المطبوخة والأسماك) |
| 10 | 9 | 8 | 7 | 6 | 5 | 4 | 3 | 2 | 1 | 0 | 6. تناول طعام طري لا يتطلب مضغًا (على سبيل المثال، البطاطس المهروسة والجيلي/الكاسترد والأطعمة المهروسة) |
| 10 | 9 | 8 | 7 | 6 | 5 | 4 | 3 | 2 | 1 | 0 | 7. أَفْتَحُ فمي على اتساع كافٍ للقضم من تفاحة كاملة |
| 10 | 9 | 8 | 7 | 6 | 5 | 4 | 3 | 2 | 1 | 0 | 8. أَفْتَحُ فمي على اتساع كافٍ لقضم ساندويتش |
| 10 | 9 | 8 | 7 | 6 | 5 | 4 | 3 | 2 | 1 | 0 | 9. أَفْتَحُ فمي على اتساع كافٍ عند التحدث |
| 10 | 9 | 8 | 7 | 6 | 5 | 4 | 3 | 2 | 1 | 0 | 10. أَفْتَحُ فمي على اتساع كافٍ للشرب من الكوب |
| 10 | 9 | 8 | 7 | 6 | 5 | 4 | 3 | 2 | 1 | 0 | 11. البلع |
| 10 | 9 | 8 | 7 | 6 | 5 | 4 | 3 | 2 | 1 | 0 | 12. التثاؤب |
| 10 | 9 | 8 | 7 | 6 | 5 | 4 | 3 | 2 | 1 | 0 | 13. الكلام |
| 10 | 9 | 8 | 7 | 6 | 5 | 4 | 3 | 2 | 1 | 0 | 14. الغناء |
| 10 | 9 | 8 | 7 | 6 | 5 | 4 | 3 | 2 | 1 | 0 | 15. يُمَكِّنُنِي وجهي من إظهار (أنني سعيد/ سعيدة) |
| 10 | 9 | 8 | 7 | 6 | 5 | 4 | 3 | 2 | 1 | 0 | 16. يُمَكِّنُنِي وجهي من إظهار (أنني غاضب/ غاضبة) |
| 10 | 9 | 8 | 7 | 6 | 5 | 4 | 3 | 2 | 1 | 0 | 17. العبوس (التكشير) |
| 10 | 9 | 8 | 7 | 6 | 5 | 4 | 3 | 2 | 1 | 0 | 18. التقبيل |
| 10 | 9 | 8 | 7 | 6 | 5 | 4 | 3 | 2 | 1 | 0 | 19. الابتسامة |
| 10 | 9 | 8 | 7 | 6 | 5 | 4 | 3 | 2 | 1 | 0 | 20. الضحك |

**Jaw Functional Limitation Scale – 20 (Back-translated version B2)**

Below is a list of activities/actions. Please indicate to what extent, during the past month, the activity/action was avoided. If it was completely avoided because it was too difficult, circle “10”. If it was avoided for reasons other than pain or difficulty, leave the item blank.

| Severe limitation | |  |  |  |  |  |  |  | No limitation | |  |
| --- | --- | --- | --- | --- | --- | --- | --- | --- | --- | --- | --- |
| 10 | 9 | 8 | 7 | 6 | 5 | 4 | 3 | 2 | 1 | 0 | 1. Chewing tough food |
| 10 | 9 | 8 | 7 | 6 | 5 | 4 | 3 | 2 | 1 | 0 | 2. Chewing hard bread |
| 10 | 9 | 8 | 7 | 6 | 5 | 4 | 3 | 2 | 1 | 0 | 3. Chewing chicken (e.g., cooked in the oven) |
| 10 | 9 | 8 | 7 | 6 | 5 | 4 | 3 | 2 | 1 | 0 | 4. Chewing biscuits |
| 10 | 9 | 8 | 7 | 6 | 5 | 4 | 3 | 2 | 1 | 0 | 5. Chewing soft food (such as pasta, canned or soft fruits, cooked vegetables, and fish) |
| 10 | 9 | 8 | 7 | 6 | 5 | 4 | 3 | 2 | 1 | 0 | 6. Eating soft food that does not require chewing (e.g., mashed potatoes, jelly/pudding, pureed food) |
| 10 | 9 | 8 | 7 | 6 | 5 | 4 | 3 | 2 | 1 | 0 | 7. I open my mouth wide enough to bite from a whole apple |
| 10 | 9 | 8 | 7 | 6 | 5 | 4 | 3 | 2 | 1 | 0 | 8. I open my mouth wide enough to bite into a sandwich |
| 10 | 9 | 8 | 7 | 6 | 5 | 4 | 3 | 2 | 1 | 0 | 9. I open my mouth wide enough when speaking |
| 10 | 9 | 8 | 7 | 6 | 5 | 4 | 3 | 2 | 1 | 0 | 10. I open my mouth wide enough to drink from a cup |
| 10 | 9 | 8 | 7 | 6 | 5 | 4 | 3 | 2 | 1 | 0 | 11. Swallowing |
| 10 | 9 | 8 | 7 | 6 | 5 | 4 | 3 | 2 | 1 | 0 | 12. Yawning |
| 10 | 9 | 8 | 7 | 6 | 5 | 4 | 3 | 2 | 1 | 0 | 13. Speaking |
| 10 | 9 | 8 | 7 | 6 | 5 | 4 | 3 | 2 | 1 | 0 | 14. Singing |
| 10 | 9 | 8 | 7 | 6 | 5 | 4 | 3 | 2 | 1 | 0 | 15. My face allows me to show that I am happy |
| 10 | 9 | 8 | 7 | 6 | 5 | 4 | 3 | 2 | 1 | 0 | 16. My face allows me to show that I am angry |
| 10 | 9 | 8 | 7 | 6 | 5 | 4 | 3 | 2 | 1 | 0 | 17. Frowning |
| 10 | 9 | 8 | 7 | 6 | 5 | 4 | 3 | 2 | 1 | 0 | 18. Kissing |
| 10 | 9 | 8 | 7 | 6 | 5 | 4 | 3 | 2 | 1 | 0 | 19. Smiling |
| 10 | 9 | 8 | 7 | 6 | 5 | 4 | 3 | 2 | 1 | 0 | 20. Laughing |
